# There is a need for a complete consideration of overall movement behaviors for the prevention, treatment, and follow-up of cancer risks and patients

**DOI:** 10.3389/fpubh.2022.1080941

**Published:** 2022-12-19

**Authors:** Gaël Ennequin, Lidia Delrieu, Adrien Rossary, Quentin Jacquinot, Fabienne Mougin, David Thivel, Martine Duclos

**Affiliations:** ^1^Université Clermont Auvergne, CRNH, AME2P, Chaire Santé en Mouvement, Clermont-Ferrand, France; ^2^Residual Tumor and Response to Treatment Laboratory, RT2Lab, Translational Research Department, INSERM, U932 Immunity and Cancer, Institute Curie, Paris University, Paris, France; ^3^Université Clermont Auvergne, INRAE, CRNH, UNH, Clermont-Ferrand, France; ^4^Regional Federative Cancer Institute of Franche-Comté, Besançon, France; ^5^Université Bourgogne Franche-Comté, EA 3920, Besançon, France; ^6^Service de Médecine du Sport et des Explorations Fonctionnelles, Centre Hospitalier Universitaire (CHU) de Clermont-Ferrand, Institut National de Recherche pour l'Agriculture, l'Alimentation et l'Environnement (INRAE), Unité de Nutrition Humaine (UNH), Centre de Recherche en Nutrition Humaine (CRNH) Auvergne, Chaire Santé en Mouvement, Université Clermont Auvergne, Clermont-Ferrand, France

**Keywords:** physical activity, sedentary behavior, cancer, prevention, treatment

## Introduction

In 2018, ~382,000 new cases of cancer (204,600 in men and 177,400 in women) and 157,400 related deaths (89,600 in men and 67,800 in women) were registered in France (1). Significantly, evidence suggests that more than 40% of cases are related to preventable risk factors (2). Environmental and lifestyle factors, including physical inactivity and sedentary behaviors (SBs), are considered to be the fourth leading risk factor for all-cause mortality worldwide (3). They have also been shown to play a major role in the incidence and recurrence of cancer as well as cancer-related mortality rates (4). Thus, faced with this immense public health issue, it is necessary to utilize preventative strategies, such as behavioral interventions, to reduce the global burden of disease associated with cancer.

## From physical activity to overall movement behavior profiles

There is no need today to further debate the beneficial role of physical activity (PA) on overall health. It is an essential and key parameter for the prevention and treatment of chronic diseases, such as cancer. PA is defined as any body movement generated by the contraction of skeletal muscle that raises energy expenditure above the resting metabolic rate and is characterized by its modality, frequency, intensity, duration, or context of practice (5). It is currently part of health recommendations for adults that encourage a minimum of 150 to 300 min per week of moderate-intensity (3.0 to <6.0 Metabolic Equivalent of Task, METs), or 75 min to 150 min per week of vigorous-intensity aerobic physical activity (≥6.0 METs), or an equivalent combination of moderate- and vigorous-intensity aerobic physical activity (MVPA) (6). In terms of primary prevention, being physically active is associated with a lower risk of many cancers. In their systematic analysis, Mctiernan et al. effectively reported a clear inverse association between PA level (PAL) and prospective risk for developing several cancers in a dose–response manner (4). According to their results, the higher the level of PA, the lower the incidence of bladder, breast, colon, endometrial, esophageal adenocarcinoma, renal, and gastric cancers for instance. Moreover, they also demonstrated a lower cancer-related mortality rate in patients respecting the health recommendations in terms of PA relative to those that did not. Among the biological mechanisms by which PA is considered to influence cancer risk are body weight management, inflammation reduction, modulation of sex and metabolic hormones, and improvement of immune function (7). Finally, PA is increasingly recognized as an effective non-pharmacological approach to counteract the adverse effects of chemotherapy and other anti-cancer treatments. To illustrate the strength of the association between PA and cancer risk, meta-analyses have clearly indicated that PA can reduce the mortality rate of breast cancer by 34%, disease recurrence by 24%, and even all-cause mortality by as much as 41% after cancer diagnosis (8). Similar results have also been described for colorectal (9) and prostate cancers (10). However, it is worth noting that patients observed less frequent PA after rather than before diagnosis (11), highlighting the physical barriers to PA for patients with cancer. The benefits of PA in tertiary prevention also extend beyond survival by increasing quality of life and reducing fatigue and secondary effects of chemotherapy, among many other benefits.

While PAL has long been studied for its role in non-communicable diseases (12), the implications of SB are increasingly being recognized. For example, the World Health Organization recommends that people must limit the amount of time spent being sedentary (13). SBs, defined as any waking behaviors characterized by an energy expenditure of ≤1.5 METs while in a sitting, reclining, or lying posture, are robustly implicated in negative health outcomes over the lifespan (14). There is also a growing body of evidence highlighting their impact on patients with chronic diseases (15). Regarding cancer, results obtained from a cohort of 8,002 adults indicated a significant association, in a dose–response manner, between the risk of cancer mortality and objectively measured sedentary time, as measured using a hip-mounted accelerometer worn for 7 consecutive days, independently of MVPA levels (16). Gathering results from 34 studies with a total of 1,331,468 participants evaluated at a high scientific standard, Patterson et al. also found strong linear associations between SB and cancer mortality (17). In their review, Jochem and collaborators highlighted a more moderate association between sedentary time and risk of colon, endometrial, and lung cancers, with limited evidence for a dose–response relation and the link with other cancer sites also limited or inconclusive (18). However, because of heterogeneity across studies in terms of sample size, type of sitting time (occupational vs. leisure-time vs. total), and potential confounders, such as body weight and methods of measures (accelerometer, questionnaires, etc.), results need to be taken with caution and further studies are needed. Blecher et al. have more recently reached similar conclusions based on a systematic approach to the available literature, suggesting that there may be some promise for intervention strategies to reduce SB in cancer patients and survivors and highlighting a need for more high-quality randomized controlled trials to understand how best to reduce SB in this population (19). Specifically, education, environmental adaptations, motivational counseling, and technologies (i.e., wearable devices or smartphones) are among the most prominent strategies that should be assessed to determine how cancer patients and survivors may experience clinical benefits through reductions in SB (19).

## Movement behavior profile and cancer

As mentioned above, the current literature to date has thoroughly investigated the relationship between PA or SB and cancer, but recent reports suggest that it is necessary to overcome oversimplified terminology such as “PA” *vs*. “SB” or “high” *vs*. “low” PA to describe the complexity of human movement behaviors. Indeed, we and other researchers encourage a move toward a multidimensional approach to PA characterization, and a detailed analysis of body motion and fine movement behaviors (decomposition, structuration, and sequencing of humans' daily movements) to identify more precisely individuals at risk for future chronic disease (20, 21). In other diseases, such as obesity, our team compared the profiles of SB time, MVPA time, and combinations of movement behavior profiles in the investigations of metabolic health. Lower sedentary time was associated with better metabolic health independently of MVPA, which might present a first step in the management of obesity when increasing MVPA is not possible (22). Thus, a better characterization of movement behavior patterns may provide a more comprehensive understanding of the interactions between the preventative potential of PA and the deleterious effects of SB on cancer risk.

Indeed, Wolvers et al. investigated the patterns of movement behaviors of 172 participants who suffered from chronic cancer-related fatigue and reported that three profiles of physical behaviors can be distinguished based on the analysis of overall PAL, MVPA, and SB, suggesting that different non-medical therapeutic strategies should be considered depending on the identified profile (23). Similarly, in a prospective cohort of 396 colorectal cancer survivors followed from 6 weeks up to 24 months after the end of the cancer treatment, Kenkhuis and collaborators assessed the longitudinal associations of SB and MVPA independently as well as their joint associations with quality of life and fatigue (24). The authors reported that decreases in SB and increases in MVPA were independently associated with improved quality of life and fatigue. However, low prolonged SB and high MVPA together demonstrated the strongest positive association with increasing quality of life and decreasing fatigue. Finally, a recent prospective study including 1,535 cancer survivors investigated the joint association of SB and PA assessed by the questionnaire (25). The results revealed that the combination of prolonged sitting with a lack of PA was highly prevalent and associated with increased all-cause and cancer-related mortality risks. Overall, these results reinforce the necessity of more precise assessments of movement behavior profiles, which could facilitate a shift toward personalized medicine in cancer patients and survivors.

## Research gaps and perspectives

To address these substantial research gaps, we have formulated recommendations for future research (Table 1). First, we encourage large cohort studies to be conducted with device-based measures of SB/PA profiles that assess their associations with incidence, recurrence, and mortality related to cancer at all sites in primary prevention. This would also help to reliably characterize the dose–response relationship between SB/PA and the risk of cancer at different sites. Similarly, post-treatment assessment of SB and PA and defining appropriate movement behavior profiles should be encouraged during the follow-up of cancer survivors. Indeed, a significant decrease in PA levels can be observed following cancer diagnosis and can last for more than 2 years (24). Additionally, it has been observed that a greater decrease in PA is observed among patients with heavier treatments (i.e., radiation and chemotherapy *vs*. surgery only) (26). Thus, physical inactivity and SB could be considered as a potential long-term consequence of cancer, especially in cancer survivors who underwent heavy treatments. Moreover, as studies exhibit methodological discrepancies (e.g., questionnaires, accelerometers, etc.), more consistent and standardized methods are needed when investigating SB and PA. A focused analysis of motion to detect minimal movements (i.e., sit-to-stand transition, leg/body displacement, and very low-intensity exercises) may provide additional information about the functional decline and identify individuals at risk following a cancer diagnosis. As such, it has been reported that breaking sitting time improves the metabolic risk factors in the general population even after adjusting for total SB and MVPA time (27), and recent findings further suggest that interrupting sedentary time may represent an opportunity to mitigate the risk of cancer mortality (16). Commercialized movement trackers have shown satisfactory acceptability in capturing the daily routine of individuals. We suggest building on this platform by developing more complex and sophisticated algorithms to better identify and refine movement behavior profiles. This could help to assess the implications of PA/SB for the evolution of the disease and long-term clinical outcomes. Furthermore, exercise prescriptions adapted to the functional state of the patient and interventions focusing on SB reduction through education and identification of barriers to PA could be improved and refined (ideally on a daily basis). In the time of precision medicine, characterizing people in terms of movement behavior profile over the lifespan could help to determine individuals at risk for cancer occurrence, recurrence, and death.

**Table 1:** Research gaps and recommendations.

**Research gaps and recommendations**
To collect data with validated devices targeting movement behavior patterns.
To develop more complex and sophisticated algorithms to better identify and refine movement behavior patterns.
To use a multidimensional approach to PA and to decipher the relationship between PA and SB, and cancer occurrence, recurrence, and related mortality.
Insufficient available evidence to fully describe the dose–response relationship (as threshold values) between PA and cancer occurrence, recurrence, and mortality.
Insufficient available evidence on all the components of movement behavior pattern such as minimal movements (i.e., sit-to-stand transition) and their association with cancer occurrence, recurrence, and mortality.
To investigate the modifications of PA and SB and their associations with cancer severity (primary prevention), types of cancer treatment and to determine whether PA and SB impairments can be considered as long-term consequences/after-effects in cancer survivors (secondary prevention) and risk factor for recurrence (tertiary prevention).
Educate and identify solutions to overcome physical activity barriers through a multidisciplinary approach and increase adhesion to PA improvements and SB limitations.
Assess PA, SB, and overall movement behavior profile during cancer follow-up up to 5 years and beyond.

## Author contributions

GE, DT, and MD conceived the idea, drafted, revised, and edited the manuscript. LD, AR, QJ, and FM revised the manuscript. All authors read and approved the final version of the article.
